# Approximation properties of *λ*-Bernstein operators

**DOI:** 10.1186/s13660-018-1653-7

**Published:** 2018-03-16

**Authors:** Qing-Bo Cai, Bo-Yong Lian, Guorong Zhou

**Affiliations:** 1 School of Mathematics and Computer Science, Quanzhou Normal University, Quanzhou, China; 2 Department of Mathematics, Yang-En University, Quanzhou, China; 3 School of Applied Mathematics, Xiamen University of Technology, Xiamen, China

**Keywords:** 41A10, 41A25, 41A36, *λ*-Bernstein operators, Bézier basis functions, Modulus of continuity, Lipschitz continuous functions, Voronovskaja asymptotic formula

## Abstract

In this paper, we introduce a new type *λ*-Bernstein operators with parameter $\lambda\in[-1,1]$, we investigate a Korovkin type approximation theorem, establish a local approximation theorem, give a convergence theorem for the Lipschitz continuous functions, we also obtain a Voronovskaja-type asymptotic formula. Finally, we give some graphs and numerical examples to show the convergence of $B_{n,\lambda }(f;x)$ to $f(x)$, and we see that in some cases the errors are smaller than $B_{n}(f)$ to *f*.

## Introduction

In 1912, Bernstein [1] proposed the famous polynomials called nowadays Bernstein polynomials to prove the Weierstrass approximation theorem as follows:

1 $$ B_{n}(f;x)=\sum_{k=0}^{n}f \biggl(\frac{k}{n} \biggr)b_{n,k}(x), $$

where $x\in[0,1]$, $n=1,2,\ldots$ , and Bernstein basis functions $b_{n,k}(x)$ are defined as:

2 $$ b_{n,k}(x)=\left ( \textstyle\begin{array}{@{}c@{}} n\\ k \end{array}\displaystyle \right )x^{k}(1-x)^{n-k}. $$

Based on this, there are many papers about Bernstein type operators [2–9]. In 2010, Ye et al. [10] defined new Bézier bases with shape parameter *λ* by

3 $$ \textstyle\begin{cases} \tilde{b}_{n,0}(\lambda;x) =b_{n,0}(x)-\frac{\lambda }{n+1}b_{n+1,1}(x), \\ \tilde{b}_{n,i}(\lambda;x) =b_{n,i}(x)+\lambda (\frac {n-2i+1}{n^{2}-1}b_{n+1,i}(x)-\frac{n-2i-1}{n^{2}-1}b_{n+1,i+1}(x) ) \quad (1\leq i\leq n-1), \\ \tilde{b}_{n,n}(\lambda;x) =b_{n,n}(x)-\frac{\lambda}{n+1}b_{n+1,n}(x), \end{cases} $$

where $\lambda\in[-1,1]$. When $\lambda=0$, they reduce to (2). It must be pointed out that we have more modeling flexibility when adding the shape parameter *λ*.

In this paper, we introduce the new *λ*-Bernstein operators,

4 $$ B_{n,\lambda}(f;x)=\sum_{k=0}^{n} \tilde{b}_{n,k}(\lambda;x)f \biggl(\frac{k}{n} \biggr), $$

where $\tilde{b}_{n,k}(\lambda;x)$ ($k=0,1,\ldots,n$) are defined in (3) and $\lambda\in[-1,1]$.

This paper is organized as follows: In the following section, we estimate the moments and central moments of these operators (4). In Sect. 3, we investigate a Korovkin approximation theorem, establish a local approximation theorem, give a convergence theorem for the Lipschitz continuous functions, and obtain a Voronovskaja-type asymptotic formula. In Sect. 4, we give some graphs and numerical examples to show the convergence of $B_{n,\lambda}(f;x)$ to $f(x)$ with different parameters.

## Some preliminary results

### Lemma 2.1

*For* *λ*-*Bernstein operators*, *we have the following equalities*:

5 $$\begin{aligned}& B_{n,\lambda}(1;x) = 1; \end{aligned}$$

6 $$\begin{aligned}& B_{n,\lambda}(t;x) = x+\frac{1-2x+x^{n+1}-(1-x)^{n+1}}{n(n-1)}\lambda ; \end{aligned}$$

7 $$\begin{aligned}& B_{n,\lambda}\bigl(t^{2};x\bigr) = x^{2}+ \frac{x(1-x)}{n}+\lambda \biggl[\frac {2x-4x^{2}+2x^{n+1}}{n(n-1)}+\frac{x^{n+1}+(1-x)^{n+1}-1}{n^{2}(n-1)} \biggr]; \end{aligned}$$

8 $$\begin{aligned}& B_{n,\lambda}\bigl(t^{3};x\bigr) = x^{3}+ \frac{3x^{2}(1-x)}{n}+\frac {2x^{3}-3x^{2}+x}{n^{2}}+\lambda\biggl[\frac{-6x^{3}+6x^{n+1}}{n^{2}}+ \frac {3x^{2}-3x^{n+1}}{n(n-1)} \\& \hphantom{B_{n,\lambda}\bigl(t^{3};x\bigr) ={}}{}+\frac{-9x^{2}+9x^{n+1}}{n^{2}(n-1)}+\frac {-4x+4x^{n+1}}{n^{3}(n-1)}+\frac{ (1-x^{n+1}-(1-x)^{n+1} )(n+3)}{n^{3} (n^{2}-1 )} \biggr]; \end{aligned}$$

9 $$\begin{aligned}& B_{n,\lambda}\bigl(t^{4};x\bigr) = x^{4}+ \frac{6 (x^{3}-x^{4} )}{n}+\frac {7x^{2}-18x^{3}+11x^{4}}{n^{2}}+\frac{x-7x^{2}+12x^{3}-6x^{4}}{n^{3}} \\& \hphantom{B_{n,\lambda}\bigl(t^{4};x\bigr) ={}}{}+\biggl[\frac{6x^{2}-2x^{3}-8x^{4}+4x^{n+1}}{n^{2}}+\frac {-x^{2}-32x^{3}+16x^{4}+17x^{n+1}}{n^{3}}+\frac{x-x^{n+1}}{n^{4}} \\& \hphantom{B_{n,\lambda}\bigl(t^{4};x\bigr) ={}}{} +\frac{7x^{2}-7x^{n+1}}{n^{2}(n-1)}+\frac {x-23x^{2}+22x^{n+1}}{n^{3}(n-1)}+\frac{(1-x)^{n+1}+x-1}{n^{4}(n-1)} \biggr]\lambda. \end{aligned}$$

### Proof

From (4), it is easy to prove $\sum_{k=0}^{n}\tilde {b}_{n,k}(\lambda;x)=1$, then we can obtain (5). Next,

$$\begin{aligned}& B_{n,\lambda}(t;x) \\& \quad = \sum_{k=0}^{n}\frac{k}{n} \tilde{b}_{n,k}(\lambda;x) \\& \quad = \sum_{k=0}^{n-1}\frac{k}{n} \biggl[b_{n,k}(x)+\lambda \biggl(\frac {n-2k+1}{n^{2}-1}b_{n+1,k}(x)- \frac{n-2k-1}{n^{2}-1}b_{n+1,k+1}(x) \biggr) \biggr] \\& \qquad {} +b_{n,n}(x)-\frac{\lambda}{n+1}b_{n+1,n}(x) \\& \quad = \sum_{k=0}^{n}\frac{k}{n}b_{n,k}(x)+ \lambda \Biggl(\sum_{k=0}^{n} \frac {k}{n}\frac{n-2k+1}{n^{2}-1}b_{n+1,k}(x)-\sum _{k=1}^{n-1}\frac{k}{n}\frac {n-2k-1}{n^{2}-1}b_{n+1,k+1}(x) \Biggr), \end{aligned}$$

as is well known, the Bernstein operators (1) preserve linear functions, that is to say, $B_{n}(at+b;x)=ax+b$. We denote the latter two parts in the bracket of the last formula by $\triangle_{1}(n;x)$ and $\triangle_{2}(n;x)$, then we have

10 $$ B_{n,\lambda}(t;x) = x+\lambda \bigl(\triangle_{1}(n;x)+\triangle _{2}(n;x) \bigr). $$

Now, we will compute $\triangle_{1}(n;x)$ and $\triangle_{2}(n;x)$,

11 $$\begin{aligned} \triangle_{1}(n;x) =&\sum_{k=0}^{n} \frac{k}{n}\frac {n-2k+1}{n^{2}-1}b_{n+1,k}(x) \\ =&\frac{1}{n-1}\sum_{k=0}^{n} \frac{k}{n}b_{n+1,k}(x)-\frac {2}{n^{2}-1}\sum _{k=0}^{n}\frac{k^{2}}{n}b_{n+1,k}(x) \\ =&\frac{(n+1)x}{n(n-1)}\sum_{k=0}^{n-1}b_{n,k}(x)- \frac{2x^{2}}{n-1}\sum_{k=0}^{n-2}b_{n-1,k}(x)- \frac{2x}{n(n-1)}\sum_{k=0}^{n-1}b_{n,k}(x) \\ =&\frac{(n+1)x}{n(n-1)} \bigl(1-x^{n} \bigr)-\frac{2x^{2}}{n-1} \bigl(1-x^{n-1} \bigr)-\frac{2x}{n(n-1)} \bigl(1-x^{n} \bigr) \\ =&\frac{x}{n}-\frac{2x^{2}}{n-1}+\frac{x^{n+1}}{n}+ \frac {2x^{n+1}}{n(n-1)}, \end{aligned}$$

and

12 $$\begin{aligned} \triangle_{2}(n;x) =&-\sum_{k=1}^{n-1} \frac{k}{n}\frac {n-2k-1}{n^{2}-1}b_{n+1,k+1}(x) \\ =&-\frac{x}{n}\sum_{k=1}^{n-1}b_{n,k}(x)+ \frac{1}{n(n+1)}\sum_{k=1}^{n-1}b_{n+1,k+1}(x)+ \frac{2x^{2}}{n-1}\sum_{k=0}^{n-2}b_{n-1,k}(x) \\ &{}-\frac{2x}{n(n-1)}\sum_{k=1}^{n-1}b_{n,k}(x)+ \frac{2}{n(n^{2}-1)}\sum_{k=1}^{n-1}b_{n+1,k+1}(x) \\ =&-\frac{x [1-(1-x)^{n}-x^{n} ]}{n}+\frac{ [1-(1-x)^{n+1}-(n+1)x(1-x)^{n}-x^{n+1} ]}{n(n+1)} \\ &{}+\frac{2x^{2} (1-x^{n-1} )}{n-1}-\frac{2x [1-(1-x)^{n}-x^{n} ]}{n(n-1)} \\ &{}+\frac{2 [1-(1-x)^{n+1}-(n+1)x(1-x)^{n}-x^{n+1} ]}{n(n^{2}-1)} \\ =&\frac{2x^{2}-x-x^{n+1}}{n-1}+\frac{1-(1-x)^{n+1}-x}{n(n-1)}. \end{aligned}$$

Combining (10), (11) and (12), we have

$$ B_{n,\lambda}(t;x)=x+\frac{1-2x+x^{n+1}-(1-x)^{n+1}}{n(n-1)}\lambda. $$

Hence, (6) is proved. Finally, by (4), we have

$$ \begin{aligned} &B_{n,\lambda}\bigl(t^{2};x\bigr) \\ &\quad = \sum_{k=0}^{n}\frac{k^{2}}{n^{2}} \tilde{b}_{n,k}(\lambda;x) \\ &\quad = \sum_{k=0}^{n-1}\frac{k^{2}}{n^{2}} \biggl[b_{n,k}(x)+\lambda \biggl(\frac {n-2k+1}{n^{2}-1}b_{n+1,k}(x)- \frac{n-2k-1}{n^{2}-1}b_{n+1,k+1}(x) \biggr) \biggr] \\ &\qquad {} +b_{n,n}(x)-\frac{\lambda}{n+1}b_{n+1,n}(x) \\ &\quad = \sum_{k=0}^{n}\frac{k^{2}}{n^{2}}b_{n,k}(x)+ \lambda \Biggl(\sum_{k=0}^{n} \frac{k^{2}}{n^{2}}\frac{n-2k+1}{n^{2}-1}b_{n+1,k}(x)-\sum _{k=1}^{n-1}\frac{k^{2}}{n^{2}}\frac{n-2k-1}{n^{2}-1}b_{n+1,k+1}(x) \Biggr), \end{aligned} $$

since $B_{n}(t^{2};x)=\sum_{k=0}^{n}\frac{k^{2}}{n^{2}}b_{n,k}(x)=x^{2}+\frac {x(1-x)}{n}$, and we denote the latter two parts in the bracket of last formula by $\triangle_{3}(n;x)$ and $\triangle_{4}(n;x)$, then we have

13 $$ B_{n,\lambda}\bigl(t^{2};x\bigr)=x^{2}+\frac{x(1-x)}{n}+ \lambda \bigl(\triangle _{3}(n;x)+\triangle_{4}(n;x) \bigr). $$

On the one hand,

14 $$\begin{aligned} \triangle_{3}(n;x) =&\sum_{k=0}^{n} \frac{k^{2}}{n^{2}}\frac{n-2k+1}{n^{2}-1}b_{n+1,k}(x) \\ =&\frac{1}{n-1}\sum_{k=0}^{n} \frac{k^{2}}{n^{2}}b_{n+1,k}(x)-\frac {2}{n^{2}-1}\sum _{k=0}^{n}\frac{k^{3}}{n^{2}}b_{n+1,k}(x) \\ =&\frac{(n+1)x^{2}}{n(n-1)}\sum_{k=0}^{n-2}b_{n-1,k}(x)+ \frac {(n+1)x}{n^{2}(n-1)}\sum_{k=0}^{n-1}b_{n,k}(x)- \frac{2x^{3}}{n}\sum_{k=0}^{n-3}b_{n-2,k}(x) \\ &{}-\frac{6x^{2}}{n(n-1)}\sum_{k=0}^{n-2}b_{n-1,k}(x)- \frac {2x}{n^{2}(n-1)}\sum_{k=0}^{n-1}b_{n,k}(x) \\ =&\frac{(n+1)x^{2} (1-x^{n-1} )}{n(n-1)}+\frac{(n+1)x (1-x^{n} )}{n^{2}(n-1)}-\frac{2x^{3} (1-x^{n-2} )}{n} \\ &{}-\frac{6x^{2} (1-x^{n-1} )}{n(n-1)}-\frac{2x (1-x^{n} )}{n^{2}(n-1)} \\ =&\frac{2x^{n+1}-2x^{3}}{n}+\frac{x^{2}-x^{n+1}}{n-1}+\frac {x-5x^{2}+4x^{n+1}}{n(n-1)}+ \frac{x^{n+1}-x}{n^{2}(n-1)}. \end{aligned}$$

On the other hand,

15 $$\begin{aligned} \triangle_{4}(n;x) =&-\sum_{k=1}^{n-1} \frac{k^{2}}{n^{2}}\frac {n-2k-1}{n^{2}-1}b_{n+1,k+1}(x) \\ =&-\frac{1}{n+1}\sum_{k=1}^{n-1} \frac{k^{2}}{n^{2}}b_{n+1,k+1}(x)+\frac {2}{n^{2}-1}\sum _{k=1}^{n-1}\frac{k^{3}}{n^{2}}b_{n+1,k+1}(x) \\ =&-\frac{x^{2}}{n}\sum_{k=0}^{n-2}b_{n-1,k}(x)+ \frac{x}{n^{2}}\sum_{k=1}^{n-1}b_{n,k}(x)- \frac{1}{n^{2}(n+1)}\sum_{k=1}^{n-1}b_{n+1,k+1}(x) \\ &{}+\frac{2x^{3}}{n}\sum_{k=0}^{n-3}b_{n-2,k}(x)+ \frac{2x}{n^{2}(n-1)}\sum_{k=1}^{n-1}b_{n,k}(x)- \frac{2}{n^{2} (n^{2}-1 )}\sum_{k=1}^{n-1}b_{n+1,k+1}(x) \\ =&-\frac{x^{2} (1-x^{n-1} )}{n}+\frac{x [1-(1-x)^{n}-x^{n} ]}{n^{2}} \\ &{}-\frac{1-(1-x)^{n+1}-(n+1)x(1-x)^{n}-x^{n+1}}{n^{2}(n+1)} \\ &{}+\frac{2x^{3} (1-x^{n-2} )}{n}+\frac{2x [1-(1-x)^{n}-x^{n} ]}{n^{2}(n-1)} \\ &{}-\frac{2 [1-(1-x)^{n+1}-(n+1)x(1-x)^{n}-x^{n+1} ]}{n^{2} (n^{2}-1 )} \\ =&\frac{(2x-1)x^{2}}{n}+\frac{x}{n(n-1)}+\frac {x-1+(1-x)^{n+1}}{n^{2}(n-1)}- \frac{x^{n+1}}{n-1}. \end{aligned}$$

Combining (13), (14) and (15), we obtain

$$ B_{n,\lambda}\bigl(t^{2};x\bigr) = x^{2}+ \frac{x(1-x)}{n}+\lambda \biggl[\frac {2x-4x^{2}+2x^{n+1}}{n(n-1)}+\frac{x^{n+1}+(1-x)^{n+1}-1}{n^{2}(n-1)} \biggr], $$

therefore, we get (7). Thus, Lemma 2.1 is proved.

Similarly, we can obtain (8) and (9) by some computations, here we omit these. □

### Corollary 2.2

*For fixed* $x\in[0,1]$ *and* $\lambda\in[-1,1]$, *using Lemma *2.1 *and by some easy computations*, *we have*

16 $$\begin{aligned}& B_{n,\lambda}(t-x;x) \\& \quad = \frac{1-2x+x^{n+1}-(1-x)^{n+1}}{n(n-1)}\lambda\leq\frac {1+2x+x^{n+1}+(1-x)^{n+1}}{n(n-1)}:=\phi_{n}(x); \end{aligned}$$

17 $$\begin{aligned}& B_{n,\lambda} \bigl((t-x)^{2};x \bigr) \\& \quad = \frac{x(1-x)}{n}+ \biggl[\frac {2x(1-x)^{n+1}+2x^{n+1}-2x^{n+2}}{n(n-1)}+\frac {x^{n+1}+(1-x)^{n+1}-1}{n^{2}(n-1)} \biggr] \lambda \\& \quad \leq \frac{x(1-x)}{n}+\frac {2x(1-x)^{n+1}+2x^{n+1}+2x^{n+2}}{n(n-1)}+\frac {x^{n+1}+(1-x)^{n+1}+1}{n^{2}(n-1)}:= \psi_{n}(x); \end{aligned}$$

18 $$\begin{aligned}& \lim_{n\rightarrow\infty}nB_{n,\lambda}(t-x;x)=0; \end{aligned}$$

19 $$\begin{aligned}& \lim_{n\rightarrow\infty}nB_{n,\lambda} \bigl((t-x)^{2};x \bigr)=x(1-x),\quad x\in(0,1); \end{aligned}$$

20 $$\begin{aligned}& \lim_{n\rightarrow\infty}n^{2}B_{n,\lambda} \bigl((t-x)^{4};x \bigr)=3x^{2}-6x^{3}+3x^{4}+6 \bigl(x^{2}-x^{3} \bigr)\lambda, \quad x\in(0,1). \end{aligned}$$

### Remark 2.3

For $\lambda\in[-1,1]$, $x\in[0,1]$, *λ*-Bernstein operators possess the endpoint interpolation property, that is,

21 $$ B_{n,\lambda}(f;0)=f(0),\qquad B_{n,\lambda}(f;1)=f(1). $$

### Proof

We can obtain (21) easily by using the definition of *λ*-Bernstein operators (4) and

$$ \tilde{b}_{n,k}(\lambda;0)= \textstyle\begin{cases} 0& (k\neq0), \\ 1 &(k=0), \end{cases}\displaystyle \qquad \tilde{b}_{n,k}(\lambda;1)= \textstyle\begin{cases} 0 &(k\neq n),\\ 1 &(k=n). \end{cases} $$

Remark 2.3 is proved. □

### Example 2.4

**Figure 1:**
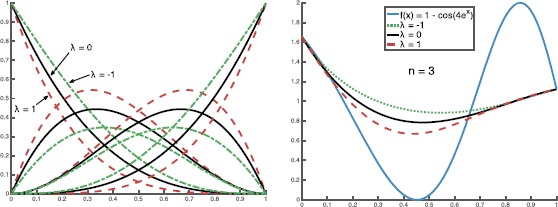
The graphs of $\tilde{b}_{3,k}(\lambda;x)$ with different values of *λ* (left) and their corresponding $B_{3,\lambda}(f;x)$ (right)

The graphs of $\tilde{b}_{3,k}(\lambda;x)$ with $\lambda= -1, 0, -1$ are shown in Fig. 1(left). The corresponding $B_{3,\lambda}(f;x)$ with $f(x) = 1 - \cos(4e^{x})$ are shown in Fig. 1(right). The graphs show the *λ*-Bernstein operators’ endpoint interpolation property, which is based on the interpolation property of $\tilde{b}_{n,k}(\lambda, x)$.

## Convergence properties

As we know, the space $C{[0,1]}$ of all continuous functions on $[0,1]$ is a Banach space with sup-norm $\|f\|:=\sup_{x\in[0,1]}|f(x)|$. Now, we give a Korovkin type approximation theorem for $B_{n,\lambda}(f;x)$.

### Theorem 3.1

*For* $f\in C{[0,1]}$, $\lambda\in[-1,1]$, *λ*-*Bernstein operators* $B_{n,\lambda}(f;x)$ *converge uniformly to* *f* *on* $[0,1]$.

### Proof

By the Korovkin theorem it suffices to show that

$$ \lim_{n\rightarrow\infty} \bigl\Vert B_{n,\lambda} \bigl(t^{i};x \bigr)-x^{i} \bigr\Vert =0, \quad i=0,1,2. $$

We can obtain these three conditions easily by (5), (6) and (7) of Lemma 2.1. Thus the proof is completed. □

The Peetre *K*-functional is defined by $K_{2}(f;\delta):=\inf_{g\in C ^{2}{[0,1]}}\{\|f-g\|+\delta\|g''\|\}$, where $\delta>0$ and $C^{2}{[0,1]}:=\{g\in C{[0,1]}: g', g''\in C{[0,1]}\}$. By [11], there exists an absolute constant $C>0$ such that

22 $$ K_{2}(f;\delta)\leq C\omega_{2} (f;\sqrt{\delta} ), $$

where $\omega_{2}(f;\delta):=\sup_{0< h\leq\delta}\sup_{x,x+h,x+2h\in [0,1]}|f(x+2h)-2f(x+h)+f(x)|$ is the second order modulus of smoothness of $f\in C{[0,1]}$. We also denote the usual modulus of continuity of $f\in C{[0,1]}$ by $\omega(f;\delta):=\sup_{0< h\leq\delta}\sup_{x,x+h\in [0,1]}|f(x+h)-f(x)|$.

Next, we give a direct local approximation theorem for the operators $B_{n,\lambda}(f;x)$.

### Theorem 3.2

*For* $f\in C{[0,1]}$, $\lambda\in[-1,1]$, *we have*

23 $$ \bigl\vert B_{n,\lambda}(f;x)-f(x) \bigr\vert \leq C\omega_{2} \bigl(f;\sqrt{\phi _{n}(x)+\psi_{n}(x)}/2 \bigr)+\omega \bigl(f;\phi_{n}(x) \bigr), $$

*where* *C* *is a positive constant*, $\phi_{n}(x)$ *and* $\psi_{n}(x)$ *are defined in* (16) *and* (17).

### Proof

We define the auxiliary operators

24 $$ \widetilde{B}_{n,\lambda}(f;x)=B_{n,\lambda}(f;x)-f \biggl(x+ \frac {1-2x+x^{n+1}-(1-x)^{n+1}}{n(n-1)}\lambda \biggr)+f(x). $$

From (5) and (6), we know that the operators $\widetilde{B}_{n,\lambda}(f;x)$ are linear and preserve the linear functions:

25 $$ \widetilde{B}_{n,\lambda}(t-x;x)=0. $$

Let $g\in C^{2}{[0,1]}$, by Taylor’s expansion,

$$ g(t)=g(x)+g'(x) (t-x)+ \int_{x}^{t}(t-u)g''(u) \,du, $$

and (25), we get

$$ \widetilde{B}_{n,\lambda}(g;x)=g(x)+\widetilde{B}_{n,\lambda} \biggl( \int _{x}^{t}(t-u)g''(u) \,du;x \biggr). $$

Hence, by (24) and (17), we have

$$\begin{aligned}& \bigl\vert \widetilde{B}_{n,\lambda}(g;x)-g(x) \bigr\vert \\& \quad \leq \biggl\vert \int_{x}^{x+\frac{1-2x+x^{n+1}-(1-x)^{n+1}}{n(n-1)}\lambda } \biggl(x+\frac{1-2x+x^{n+1}-(1-x)^{n+1}}{n(n-1)}\lambda-u \biggr)g''(u)\,du \biggr\vert \\& \qquad {} + \biggl\vert B_{n,\lambda} \biggl( \int_{x}^{t}(t-u)g''(u) \,du;x \biggr) \biggr\vert \\& \quad \leq \int_{x}^{x+\frac{1-2x+x^{n+1}-(1-x)^{n+1}}{n(n-1)}\lambda} \biggl\vert x+\frac{1-2x+x^{n+1}-(1-x)^{n+1}}{n(n-1)} \lambda-u \biggr\vert \bigl\vert g''(u) \bigr\vert \,du \\& \qquad {} +B_{n,\lambda} \biggl( \biggl\vert \int_{x}^{t}(t-u) \bigl\vert g''(u) \bigr\vert \,du \biggr\vert ;x \biggr) \\& \quad \leq \biggl[B_{n,\lambda} \bigl((t-x)^{2};x \bigr)+ \frac {1+2x+x^{n+1}+(1-x)^{n+1}}{n(n-1)} \biggr] \bigl\Vert g'' \bigr\Vert \\& \quad \leq \bigl[\phi_{n}(x)+\psi_{n}(x) \bigr] \bigl\Vert g'' \bigr\Vert . \end{aligned}$$

On the other hand, by (24), (5) and (4), we have

26 $$ \bigl\vert \widetilde{B}_{n,\lambda}(f;x) \bigr\vert \leq \bigl\vert B_{n,\lambda }(f;x) \bigr\vert +2\|f\|\leq\|f\|B_{n,\lambda}(1;x)+2\|f\| \leq3\|f\|. $$

Now, (24) and (26) imply

$$\begin{aligned} \bigl\vert B_{n,\lambda}(f;x)-f(x) \bigr\vert \leq& \bigl\vert \widetilde{B}_{n,\lambda}(f-g;x)-(f-g) (x) \bigr\vert + \bigl\vert \widetilde{B}_{n,\lambda}(g;x)-g(x) \bigr\vert \\ &{}+ \biggl\vert f \biggl(x+\frac{1-2x+x^{n+1}-(1-x)^{n+1}}{n(n-1)}\lambda \biggr)-f(x) \biggr\vert \\ \leq&4 \Vert f-g \Vert + \bigl[\phi_{n}(x)+\psi_{n}(x) \bigr] \bigl\Vert g'' \bigr\Vert +\omega \bigl(f; \phi_{n}(x) \bigr). \end{aligned}$$

Hence, taking infimum on the right hand side over all $g\in C^{2}{[0,1]}$, we get

$$ \bigl\vert B_{n,\lambda}(f;x)-f(x) \bigr\vert \leq4K_{2} \biggl(f;\frac{\phi _{n}(x)+\psi_{n}(x)}{4} \biggr)+\omega \bigl(f;\phi_{n}(x) \bigr). $$

By (22), we have

$$ \bigl\vert B_{n,\lambda}(f;x)-f(x) \bigr\vert \leq C\omega_{2} \bigl(f;\sqrt{\phi _{n}(x)+\psi_{n}(x)}/2 \bigr)+\omega \bigl(f;\phi_{n}(x) \bigr), $$

where $\phi_{n}(x)$ and $\psi_{n}(x)$ are defined in (16) and (17). This completes the proof of Theorem 3.2. □

### Remark 3.3

For any $x\in[0,1]$, we have $\lim_{n\rightarrow\infty}\phi_{n}(x)=0$ and $\lim_{n\rightarrow\infty}\psi_{n}(x)=0$, these give us a rate of pointwise convergence of the operators $B_{n,\lambda}(f;x)$ to $f(x)$.

Now, we study the rate of convergence of the operators $B_{n,\lambda }(f;x)$ with the help of functions of Lipschitz class $\operatorname{Lip}_{M}(\alpha )$, where $M>0$ and $0<\alpha\leq1$. A function *f* belongs to $\operatorname{Lip}_{M}(\alpha)$ if

27 $$ \bigl\vert f(y)-f(x) \bigr\vert \leq M \vert y-x \vert ^{\alpha} \quad (x,y\in\mathbb{R}). $$

We have the following theorem.

### Theorem 3.4

*Let* $f\in \operatorname{Lip}_{M}(\alpha)$, $x\in[0,1]$ *and* $\lambda\in [-1,1]$, *then we have*

$$ \bigl\vert B_{n,\lambda}(f;x)-f(x) \bigr\vert \leq M \bigl[ \psi_{n}(x) \bigr]^{\frac{\alpha}{2}}, $$

*where* $\psi_{n}(x)$ *is defined in* (17).

### Proof

Since $B_{n,\lambda}(f;x)$ are linear positive operators and $f\in \operatorname{Lip}_{M}(\alpha)$, we have

$$\begin{aligned} \bigl\vert B_{n,\lambda}(f;x)-f(x) \bigr\vert \leq&B_{n,\lambda} \bigl( \bigl\vert f(t)-f(x) \bigr\vert ;x\bigr) \\ =&\sum_{k=0}^{n}\tilde{b}_{n,k}( \lambda;x) \biggl\vert f \biggl(\frac {k}{n} \biggr)-f(x) \biggr\vert \\ \leq&M\sum_{k=0}^{n}\tilde{b}_{n,k}( \lambda;x) \biggl\vert \frac {k}{n}-x \biggr\vert ^{\alpha} \\ \leq&M\sum_{k=0}^{n} \biggl[ \tilde{b}_{n,k}(\lambda;x) \biggl(\frac {k}{n}-x \biggr)^{2} \biggr]^{\frac{\alpha}{2}} \bigl[\tilde {b}_{n,k}( \lambda;x) \bigr]^{\frac{2-\alpha}{2}}. \end{aligned}$$

Applying Hölder’s inequality for sums, we obtain

$$\begin{aligned} \bigl\vert B_{n,\lambda}(f;x)-f(x) \bigr\vert \leq&M \Biggl[\sum _{k=0}^{n}\tilde{b}_{n,k}(\lambda;x) \biggl(\frac{k}{n}-x \biggr)^{2} \Biggr]^{\frac{\alpha}{2}} \Biggl[ \sum_{k=0}^{n}\tilde {b}_{n,k}( \lambda;x) \Biggr]^{\frac{2-\alpha}{2}} \\ =&M \bigl[B_{n,\lambda} \bigl((t-x)^{2};x \bigr) \bigr]^{\frac{\alpha}{2}}. \end{aligned}$$

Thus, Theorem 3.4 is proved. □

Finally, we give a Voronovskaja asymptotic formula for $B_{n,\lambda}(f;x)$.

### Theorem 3.5

*Let* $f(x)$ *be bounded on* $[0,1]$. *Then*, *for any* $x\in(0,1)$ *at which* $f''(x)$ *exists*, $\lambda\in[-1,1]$, *we have*

28 $$ \lim_{n\rightarrow\infty}n \bigl[B_{n,\lambda}(f;x)-f(x) \bigr]= \frac {f''(x)}{2} \bigl[x(1-x) \bigr]. $$

### Proof

Let $x\in[0,1]$ be fixed. By the Taylor formula, we may write

29 $$ f(t)=f(x)+f'(x) (t-x)+\frac{1}{2}f''(x) (t-x)^{2}+r(t;x) (t-x)^{2}, $$

where $r(t;x)$ is the Peano form of the remainder, $r(t;x)\in C{[0,1]}$, using L’Hopital’s rule, we have

$$\begin{aligned} \lim_{t\rightarrow x}r(t;x) =&\lim_{t\rightarrow x} \frac {f(t)-f(x)-f'(x)(t-x)-\frac{1}{2}f''(x)(t-x)^{2}}{(t-x)^{2}} \\ =&\lim_{t\rightarrow x}\frac{f'(t)-f'(x)-f''(x)(t-x)}{2(t-x)}=\lim_{t\rightarrow x} \frac{f''(t)-f''(x)}{2}=0. \end{aligned}$$

Applying $B_{n,\lambda}(f;x)$ to (29), we obtain

30 $$\begin{aligned} \lim_{n\rightarrow\infty}n \bigl[B_{n,\lambda}(f;x)-f(x) \bigr] =&f'(x)\lim_{n\rightarrow\infty}nB_{n,\lambda}(t-x;x)+ \frac {f''(x)}{2}\lim_{n\rightarrow\infty}nB_{n,\lambda} \bigl((t-x)^{2};x \bigr) \\ &{}+\lim_{n\rightarrow\infty}nB_{n,\lambda} \bigl(r(t;x) (t-x)^{2};x \bigr). \end{aligned}$$

By the Cauchy–Schwarz inequality, we have

31 $$ B_{n,\lambda} \bigl(r(t;x) (t-x)^{2};x \bigr)\leq \sqrt{B_{n,\lambda} \bigl(r^{2}(t;x);x \bigr)}\sqrt{B_{n,\lambda} \bigl((t-x)^{4};x \bigr)}, $$

since $r^{2}(x;x)=0$, then we can obtain

32 $$ \lim_{n\rightarrow\infty}nB_{n,\lambda} \bigl(r(t;x) (t-x)^{2};x \bigr)=0 $$

by (31) and (20). Finally, using (18), (19), (32) and (30), we get

$$ \lim_{n\rightarrow\infty}n \bigl[B_{n,\lambda}(f;x)-f(x) \bigr]= \frac {f''(x)}{2} \bigl[x(1-x) \bigr]. $$

Theorem 3.5 is proved. □

## Graphical and numerical analysis

In this section, we give several graphs and numerical examples to show the convergence of $B_{n,\lambda}(f;x)$ to $f(x)$ with different values of *λ* and *n*.

**Figure 2:**
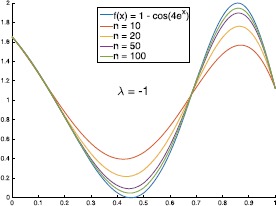
The graphs of $B_{n,-1}(f;x)$ with different values of *n*

**Figure 3:**
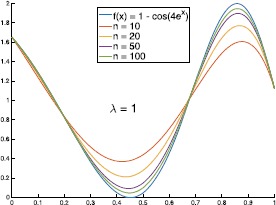
The graphs of $B_{n,1}(f;x)$ with different values of *n*

**Figure 4:**
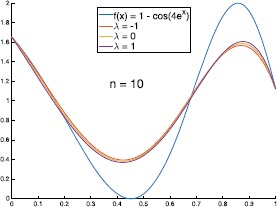
The graphs of $B_{10,\lambda}(f;x)$ with different values of *λ*

Let $f(x) = 1 - \cos(4e^{x})$, the graphs of $B_{n,-1}(f;x)$ and $B_{n,1}(f;x)$ with different values of *n* are shown in Figs. 2 and 3. In Table 1, we give the errors of the approximation of $B_{n,\lambda}(f;x)$ to $f(x)$. We can see from Table 1 that in some special cases (such as $n=10, 20$ and $\lambda>0$), the errors of $\|f-B_{n,\lambda}(f)\|_{\infty}$ are smaller than $\|f-B_{n,0}(f)\|_{\infty}$ (where $B_{n,0}(f;x)$ are classical Bernstein operators). Figure 4 shows the graphs of $B_{n,\lambda}(f;x)$ with $n = 10$ and different values of *λ*.

**Table 1:** The errors of the approximation of $B_{n,\lambda}(f;x)$ to $f(x)$ with different values of *n* and *λ*

*λ*	$\|f - B_{n,\lambda}(f)\|_{\infty}$				
	*n* = 10	*n* = 20	*n* = 50	*n* = 100	*n* = 150
−1	0.437813	0.242921	0.104883	0.054106	0.036478
−0.5	0.430221	0.241337	0.104880	0.054136	0.036496
0	0.422857	0.239850	0.104884	0.054166	0.036513
0.5	0.415719	0.238458	0.104897	0.054196	0.036531
1	0.408808	0.237158	0.104918	0.054229	0.036550
